# Changes in body weight in response to pecan-enriched diets with and without substitution instructions: a randomised, controlled trial

**DOI:** 10.1017/jns.2022.14

**Published:** 2022-03-07

**Authors:** Liana L. Guarneiri, Chad M. Paton, Jamie A. Cooper

**Affiliations:** 1 Department of Nutritional Sciences, University of Georgia, Athens, GA, USA; 2 Department of Food Science and Technology, University of Georgia, Athens, GA, USA

**Keywords:** Adiposity, DXA, Energy intake, Weight management, ADD, consumed pecans as part of a free-living diet, BF, total body fat percentage, BP, blood pressure, BW, body weight, EI, energy intake, ERS, energy report score, HC, hip circumference, MET, metabolic equivalent, SUB, substituted pecans for isocaloric foods from their habitual diet, WC, waist circumference

## Abstract

Substantial evidence suggests that regular tree nut consumption does not lead to changes in body weight (BW). However, these studies used a variety of dietary substitution instructions which may confound the interpretation of prior BW outcomes. The purpose of the present study was to examine the impact of daily pecan consumption, with or without isocaloric substitution instructions, on BW and composition. This was an 8-week randomised, controlled trial with three treatments: a nut-free control group (*n* 32) and two pecan groups. ADD (*n* 30) consumed pecans (68 g/d) as part of a free-living diet, and SUB (*n* 31) substituted the pecans (68 g/d) for isocaloric foods from their habitual diet. BW and total body fat percentage (BF) were measured, and theoretical changes in these outcomes if pecans were consumed without compensation were determined. BW increased in all groups across the intervention, and there was a trend (*P* = 0⋅09) for an increase in ADD (1⋅1 ± 0⋅2 kg) and SUB (0⋅9 ± 0⋅3 kg) compared to control (0⋅3 ± 0⋅2 kg). In addition, there was increased BF in SUB (1⋅0 ± 0⋅3 %; *P* = 0⋅005) but not ADD (0⋅1 ± 0⋅2 %) or control (−0⋅2 ± 0⋅3 %) There was a large difference in the actual *v.* theoretical change in BW regardless of pecan treatment (actual: 1⋅1 ± 0⋅2 and 0⋅9 ± 0⋅3 *v.* theoretical: 3⋅3 ± 0⋅0 and 3⋅2 ± 0⋅0 kg in ADD and SUB, respectively; *P* < 0⋅001). Furthermore, there was a difference in actual *v.* theoretical change in BF in ADD (0⋅1 ± 0⋅2 *v.* 1⋅2 ± 0⋅1 %; *P* = 0⋅002) but not SUB or control. In conclusion, daily pecan consumption for 8 weeks did not result in significant weight gain, regardless of dietary substitution instructions.

## Introduction

More than 40 % of U.S. adults have obesity, which is associated with elevated risk for chronic diseases and preventable death^(1)^. Since weight-loss interventions often result in weight regain^(2)^, promoting obesity prevention is an important approach for combatting the obesity epidemic. One method for achieving energy balance and maintaining weight is to consume nutrient-dense foods that are rich in fibre, protein and unsaturated fatty acids^(3–6)^. Tree nuts are rich sources of these nutrients, and there is substantial evidence that suggests regular tree nut consumption, even in large quantities, does not cause weight gain^(7,8)^.

Despite the promising evidence for tree nuts with respect to body weight (BW) regulation, there are methodological differences between intervention studies to consider. Some tree nut studies provide no dietary instructions^(9,10)^, while others provide instructions to substitute energy-equivalent foods or specific macronutrients in their typical diet for the nuts provided^(11,12)^. Furthermore, other studies have provided all meals in an outpatient feeding setting designed to keep participants in energy balance^(13,14)^. We recently conducted a systematic review and meta-analysis to examine the impact of the absence of dietary substitution *v.* some type of dietary substitution instructions on BW and concluded that neither condition resulted in changes in BW^(7)^.

Contrary to the result of our meta-analysis, two intervention studies involving walnuts or peanuts that directly compared changes in BW and body fat percentage (BF) in participants who received substitution instructions *v.* no instructions reported that substitution instructions impact these outcomes^(15,16)^. Njike *et al.*^(15)^ reported that a walnut diet without dietary advice increased BF, while the walnut diet with dietary advice improved waist circumference. Likewise, Alper *et al.*^(16)^ reported that participants gained BW when consuming peanuts without dietary guidance, but there was no change in BW when peanuts were substituted for other fats in the diet. Since the conclusions from our meta-analysis and these two studies conflict, further research needs to be conducted to directly compare the impact of substitution instructions on weight outcomes during trials involving nuts. The purpose of the present study was to examine the impact of daily pecan consumption, with or without isocaloric substitution instructions, for an 8-week period on BW and BF (primary outcomes) in sedentary adults. We hypothesised that the pecan group with no substitution instructions (ADD) would have an increase in BW and BF, and that the increase would be greater than SUB (pecan group with isocaloric substitution instructions) and control (no nuts) groups. We did, however, expect this increase to be less than theoretical calculations. Finally, we expected no differences in weight changes between control and SUB.

## Methods

### Study design

This was a randomised, parallel controlled trial (clinicaltrials.gov: NCT04376632) involving an 8-week intervention conducted at the University of Georgia (UGA). The participants were unaware that there were two pecan groups that received different dietary instructions. Data collection occurred from August 2018 to May 2021 when the goal of at least twenty-six subjects/groups was obtained. The protocol included a screening visit and three testing visits (baseline, 4 and 8 weeks). Subjects were randomly assigned to one of three groups: a ‘no nut’ control group or one of two pecan groups (ADD or SUB). Subjects in ADD and SUB each consumed 68 g/d of pecans for 8 weeks; however, dietary instructions for pecan incorporation differed by group. The present study was conducted according to the guidelines laid down in the Declaration of Helsinki and all procedures involving human subjects/patients were approved by the Institutional Review Board for human subjects at the UGA. Written consent was obtained from all subjects.

### Participants

One hundred twenty-four sedentary men and women between the ages of 30 and 75 years with a body mass index (BMI) of ≥18⋅5 kg/m^2^ were assessed for eligibility. Exclusion criteria included habitual nut consumption (>2 servings/week), nut allergies, special diets (i.e. ketogenic diet, intermittent fasting), excessive alcohol use (>3 drinks/d for men or >2 drinks/d for women), tobacco or nicotine use, exercise of >3 h/week, weight change of >5 % of BW in the past 3 months, history of medical events or medication use affecting digestion, absorption or metabolism, gastrointestinal surgery, and chronic or metabolic diseases. Individuals taking lipid-lowering medications, fish oil supplements, steroid/hormone therapy or medications for diabetes mellitus or attention deficit hyperactivity disorder were also excluded. Finally, individuals with the following biomarkers were excluded: fasting glucose of >7 mmol/L, fasting triacylglycerols of >4 mmol/L and blood pressure (BP) of >180/120 mmHg. Eligibility based on these biomarkers was determined from fasting measurements at the screening visit.

### Protocol

#### Screening visit

Individuals arrived at the Human Nutrition Lab (HNL) following an 8–12 h overnight fast and 24 h without exercise or alcohol. A lipid panel, glucose and anthropometrics were assessed to confirm eligibility. If individuals qualified for the study, subjects were randomised to one of the three treatment groups by a researcher that was not involved in data collection or analysis. An allocation ratio of 1:1:1, a permuted block design (balanced for age, sex and BMI) and a random number generator were used to randomise participants.

#### Pre-intervention visit (baseline visit)

Participants completed a 2-d food diary containing one weekend day and one weekday^(17)^ between the screening visit and the pre-diet intervention visit (baseline). The night before the baseline visit, participants consumed a lead-in dinner meal and snack (provided by research personnel) that contained 50 % of total energy from carbohydrate, 15 % protein and 35 % fat. For the baseline visit, participants arrived at the HNL following an 8–12 h overnight fast and 24 h without exercise or alcohol. Participants changed into a hospital gown and removed footwear for the BW measurement, which was recorded to the nearest 0⋅001 kg using a calibrated electronic scale. Next, height was measured to the nearest millimetre using a stadiometer. Waist circumference (WC) and hip circumference (HC) were measured in triplicate to the nearest mm, and BP was measured in triplicate with 30 s between each measurement. For WC, HC and BP, the average of the 3 measurements was used.

Next, body composition was measured by dual-energy X-ray absorptiometry (DXA) (Discovery A; Hologic Inc., Waltham, MA, USA). Weight, WC–HC and body composition were primary outcomes, while BP was a secondary outcome. Finally, physical activity, stress and preference for fat were evaluated via validated questionnaires (International Physical Activity Questionnaire (IPAQ) Short Form, Perceived Stress Scale (PSS) and fat preference questionnaire, respectively)^(18–20)^. To quantify the taste and frequency scores for the fat preference questionnaire, the percent of food sets in which high-fat foods were reported to ‘taste better’ and be ‘eaten more often’ were calculated^(20)^. In addition, the frequency score was subtracted from the taste score to quantify a difference score.

#### 8-week dietary intervention

The day after the baseline visit, all participants began the 8-week intervention. Written diet instructions were provided to all participants. Subjects were instructed to avoid all forms of nuts that were not part of the study and to consume ≤2 servings (64 g) of nut butter/week. Participants in ADD were provided with 68 g (~0⋅5 cup or 2⋅25 ounces) portions of pecans to consume each day as part of their free-living diet with no additional dietary instructions. Participants in SUB were instructed to substitute the 470 kcal provided by the 68 g of pecans for foods habitually consumed in their free-living diet. Trained research personnel guided the participants on how to make appropriate energy substitutions based on their previously completed food diaries. For example, if the participant habitually consumed snacks throughout the day, the research personnel highlighted the energy content of the snacks and asked the participant if it was feasible to replace the habitual snacks with the provided pecans. The guidance provided was individualised based on each participant's dietary intake. Table 1 shows the complete nutrition information for the 68 g portion of pecans. In addition, they were instructed to eat the pecans in their raw form (no roasting, cooking or baking) but could add them to other foods. Finally, all subjects were instructed to avoid consuming >42 g alcohol/d (men) or >28 g alcohol/d (women) and were asked not to make any other changes to their diet or activity levels. Participants were unaware of the diet instructions that were provided to other groups to prevent unintentional or intentional changes in behaviour.

**Table 1.** Nutrient breakdown for pecans (68 g)

Energy (kcal)	469⋅9
Carbohydrates (g)	9⋅4
Total sugars (g)	2⋅7
Total dietary fibre (g)	6⋅5
Protein (g)	6⋅2
Fat (g)	48⋅9
SFA (g)	4⋅2
MUFA (g)	27⋅7
Oleic acid	27⋅6
Paullinic acid	0⋅1
PUFA (g)	14⋅7
ALA (*n*-3)	0⋅7
Linoleic acid (*n*-6)	14⋅0

kcal, kilocalorie; g, gram; SFA, saturated fatty acid; MUFA, monounsaturated fatty acid; PUFA,  polyunsaturated fatty acid; ALA,  α-linolenic acid.

#### Weekly responsibilities

Participants in ADD and SUB also completed a daily nut compliance log that detailed the time of day for pecan consumption. Nut compliance logs were submitted to research staff once per week. Poor compliance was categorised as consumption of <75 % of pecans throughout the 8-week intervention. All participants completed a food diary once per week alternating between weekdays and weekend days. Daily nutrient intakes based on food diaries were assessed using the Food Processor SQL software (version 10.12.0). The nutrients from the two baseline food diaries, and then the food diaries from weeks 1–8, were averaged before analysis. Physical activity was assessed at baseline and during weeks 2, 4, 6 and 8. Physical activity was averaged for the weeks during the intervention before analysis.

#### Mid- and post-intervention visits (weeks 4 and 8)

After 4 and 8 weeks of the diet intervention, participants returned to the HNL under the same conditions as baseline. At both visits, participants completed the exact same procedures and measurements that took place at baseline, except the fat preference questionnaire was not evaluated at week 4. The PSS from weeks 4 and 8 was averaged together before analysis.

### Statistical analyses

SAS version 9.2 statistical package (SAS Institute Inc, Cary, NC, USA) and R version 3.6.2 (The R Foundation, Vienna, Austria) were used for statistical analyses. All hypotheses and analytic plans were pre-specified. All values were reported as mean ± sem unless otherwise noted. Statistical significance was set at *P* ≤ 0⋅05. A samples size of seventy-eight (twenty-six per group) was estimated to detect a significant difference in BW between the two pecan groups using G*power 3.19.7 assuming at least 80 % power and an *α* of 0⋅05. This calculation was based on the mean difference of 0⋅8 kg between the peanut interventions with and without substitution instructions in the study by Alper *et al.*^(16)^. The theoretical changes in BW and BF in ADD and SUB if pecans were consumed without compensation were calculated for each participant using the National Institute of Health (NIH) Body Weight Planner, which accounts for the physiological energy adaptations during periods of weight change^(21)^. The NIH Body Weight Planner was also used to calculate the estimated energy intake (EI) during the study based on each participant's weight change, age, sex and height. A measure of under- or over-reporting on food diaries (energy report score, ERS) was calculated by subtracting the estimated EI during the study based on changes in BW from the average EI reported on food diaries during the intervention. The ERS for each group was compared using a one-way analysis of variance (ANOVA).

An unpaired *t*-test was used to assess differences in nut compliance between the two pecan groups. For anthropometrics, dietary intake and questionnaires, a repeated measures linear mixed model for treatment (ADD, SUB and control) and visit (baseline, 4 and 8 weeks) was used to test for differences. In addition, a two-way ANOVA was used to test for differences in the actual *v.* theoretical change in BW and BF. *Post hoc* analyses were done using Tukey's test. Finally, exploratory multiple regression analyses were conducted to determine predictors of the change in BW and BF in the two pecan groups. Factors included in the model were treatment (0 = ADD, 1 = SUB), baseline age, sex, BW, BF, physical activity (total MET, min/week), low-density lipoprotein (LDL) (from the screening visit), fat preference difference score, sugar intake and the ERS. To determine predictors, two-way stepwise multiple regression and best subsets multiple regression approaches were employed. The two-way stepwise multiple regression analysis selected the model that minimised the akaike information criterion^(22)^. Similarly, the best subsets methods selected models that minimised the Bayesian Information Criterion (BIC) and Mallow's *C_p_*^(22–24)^. Multiple regression analysis was used to model the change in BW and BF with the predictors obtained from the model selection methods.

## Results

### Subjects

**Fig. 1.**
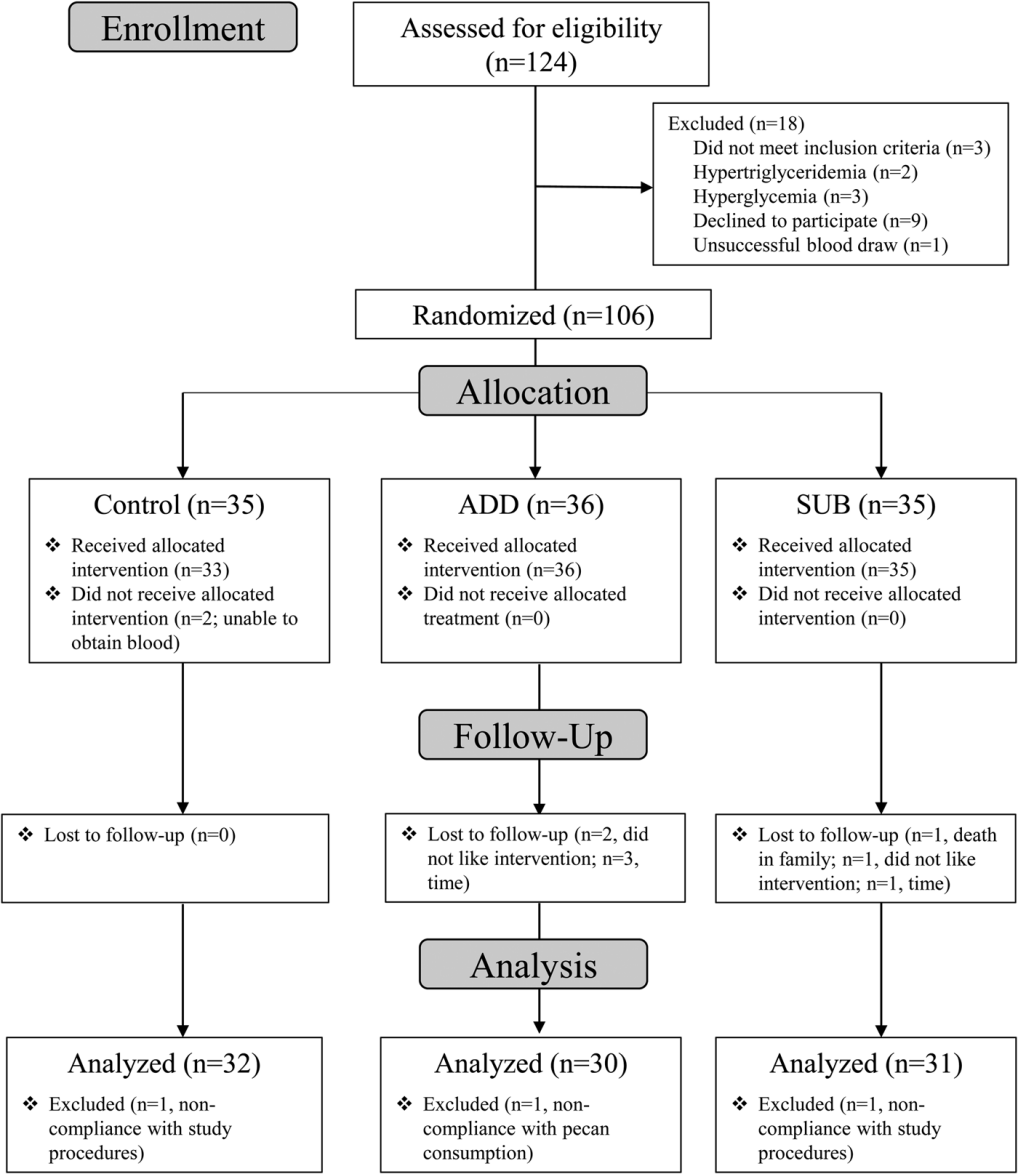
Consolidating standards of reporting (CONSORT) flow diagram selection of participants.

One hundred six participants were randomly assigned to an intervention (ADD: *n* 36, SUB: *n* 35, control: *n* 35); however, twelve participants did not start or complete the intervention and were not included in final analyses (Fig. 1). Three of these twelve participants were excluded after follow-up due to non-compliance (*n* 1 did not meet >75 % pecan compliance; *n* 2 were non-compliant with study procedures). Therefore, ninety-three participants completed the intervention (*n* 20 women and 10 men for ADD; *n* 21 women and 10 men for SUB; *n* 23 women and 9 men for control) and were included in the per-protocol analyses of primary and secondary outcomes. The average age of participants in ADD, SUB and control was 47 ± 2, 44 ± 2 and 47 ± 2 years. Participants in both ADD and SUB consumed 95 % of the pecans provided, and compliance was not different between groups. No participant reported poor compliance, and there was no report of intake of nuts in the control group according to food diaries.

### Anthropometrics and BP

Anthropometrics and BP at baseline, 4 and 8 weeks are presented in Table 2. There were no differences between groups at baseline for any outcome. For BW, there was a significant effect of visit (time) (*P* < 0⋅001), no effect of treatment (*P* = 0⋅39) and a trend for a visit by treatment interaction (*P* = 0⋅09). The *post hoc* analyses revealed that there was an increase in BW from baseline to 4 weeks (*P* = 0⋅01), baseline to 8 weeks (*P* < 0⋅001) and 4 to 8 weeks (*P* < 0⋅001) regardless of treatment. The trend for the interaction effect, however, was driven by BW changes from baseline to 8 weeks in ADD (77⋅6 ± 3⋅0 to 78⋅7 ± 3⋅1 kg; *P*=<0⋅001) and SUB (84⋅7 ± 3⋅5 to 85⋅6 ± 3⋅5 kg; *P* < 0⋅001), but not the control group (80⋅1 ± 4⋅4 to 80⋅4 ± 4⋅4 kg; *P* = 0⋅85). For BF, there was a significant visit by treatment interaction (*P* = 0⋅005) but no main effect of treatment (*P* = 0⋅35) or visit (*P* = 0⋅13). The interaction was for an increase in BF within SUB from baseline to 8 weeks (*P* = 0⋅001) with no change in either ADD or control. Similar to BW data, for BMI, there was a main effect of visit (*P* < 0⋅001) but no treatment effect (*P* = 0⋅18) or interactions (*P* = 0⋅14). The effect of visit was an increase in BMI from baseline to 4 weeks (*P* = 0⋅006), baseline to 8 weeks (*P* < 0⋅001) and 4 to 8 weeks (*P* = 0⋅008) regardless of treatment. For SBP, there was also a visit effect (*P* = 0⋅04) but no treatment effect (*P* = 0⋅70) or interactions (*P* = 0⋅63). The main effect of visit was an increase in SBP from baseline to 8 weeks only (*P* = 0⋅04) regardless of treatment. Finally, there were no main or interaction effects for WC, HC, waist-to-hip ratio or diastolic blood pressure (ns).

**Table 2.** Anthropometrics across the intervention

	ADD (*n* 30)						SUB (*n* 31)						Control (*n* 32)					
	Week 0		Week 4		Week 8		Week 0		Week 4		Week 8		Week 0		Week 4		Week 8	
	Mean	sem	Mean	sem	Mean	sem	Mean	sem	Mean	sem	Mean	sem	Mean	sem	Mean	sem	Mean	sem
Weight (kg)^a^	77⋅6	3⋅0	78⋅2	3⋅0	78⋅7^†^	3⋅1	84⋅7	3⋅5	85⋅1	3⋅5	85⋅6^†^	3⋅5	80⋅1	4⋅4	80⋅2	4⋅4	80⋅4	4⋅4
BMI (kg/m^2^)^a^	27⋅6	0⋅9	27⋅8	0⋅9	28⋅0	0⋅9	30⋅3	1⋅2	30⋅5	1⋅2	30⋅6	1⋅2	28⋅1	1⋅2	28⋅1	1⋅2	28⋅2	1⋅2
WC (cm)	89⋅0	2⋅3	88⋅9	2⋅0	88⋅9	2⋅3	93⋅7	2⋅6	94⋅1	2⋅8	94⋅4	2⋅6	89⋅0	3⋅0	89⋅4	3⋅2	89⋅8	3⋅1
HC (cm)	106⋅8	1⋅9	107⋅6	1⋅9	107⋅3	1⋅8	111⋅9	2⋅1	111⋅9	2⋅0	113⋅4	2⋅2	108⋅2	2⋅3	108⋅4	2⋅1	108⋅4	2⋅3
WHR	0⋅83	0⋅01	0⋅83	0⋅01	0⋅83	0⋅01	0⋅84	0⋅02	0⋅84	0⋅02	0⋅83	0⋅01	0⋅82	0⋅02	0⋅82	0⋅02	0⋅83	0⋅02
Body fat (%)	30⋅3	1⋅4	30⋅6	1⋅5	30⋅4	1⋅4	32⋅4	1⋅2	33⋅0	1⋅3	33⋅5*	1⋅3	31⋅1	1⋅4	30⋅8	1⋅3	30⋅8	1⋅2
SBP (mmHg)^a^	124	3	123	3	127	3	124	3	127	3	128	3	122	3	123	3	125	3
DBP (mmHg)	80	3	80	3	82	2	79	2	80	2	81	2	78	2	76	3	77	2

ADD, consumed pecans as part of a free-living diet; SUB, substituted pecans for isocaloric foods from their habitual diet; BMI, body mass index; HC, hip circumference; WC, waist circumference; WHR, waist-to-hip ratio; SBP, systolic blood pressure; DBP, diastolic blood pressure.

There were no differences between groups at baseline.

* This indicates a significant treatment by visit interaction with an increase in body fat % within the SUB group only (*P* ≤ 0⋅05).

† This indicates a trend for a treatment by visit interaction for greater increases in weight for both pecan groups compared to control (*P* < 0⋅10).

a This indicates a significant main effect of visit at *P* ≤ 0⋅05.

### Theoretical weight change

**Fig. 2.**
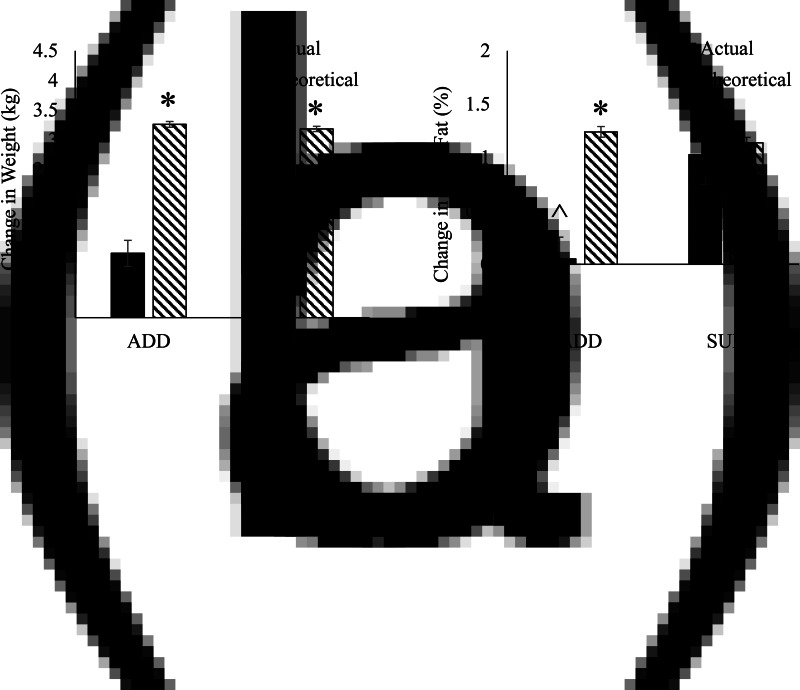
Changes in actual and theoretical (a) BW and (b) total body fat percentage in ADD and SUB from baseline to 8 weeks. A two-way analysis of variance (ANOVA) was used to test for differences. Tukey's test was used for *post hoc* analyses. (a) * indicates a significant difference between the actual and theoretical BW within ADD and SUB (*p* ≤ 0⋅05). (b) * indicates a significant difference between the actual and theoretical BF within ADD only (*p* ≤ 0⋅05); ^ indicates a significant difference between groups. BW, body weight; ADD, consumed pecans as part of a free-living diet; SUB, substituted pecans for isocaloric foods from their habitual diet.

The actual and theoretical changes in BW and BF from baseline to 8 weeks in the ADD and SUB groups are presented in Fig. 2. There was a significant effect of the type (actual *v.* theoretical) for BW (*P* < 0⋅001) but no effect of treatment (*P* = 0⋅53) or a type by treatment interaction (*P* < 0⋅001). The significant main effect was for a difference between actual *v.* theoretical changes in BW in both pecan groups (actual: 1⋅1 ± 0⋅2 and 0⋅9 ± 0⋅3 *v.* theoretical: 3⋅3 ± 0⋅0 and 3⋅2 ± 0⋅0 kg in ADD and SUB, respectively; *P* < 0⋅001) (Fig. 2(a)). Furthermore, there was a main effect of treatment (*P* = 0⋅01) and type (*P* < 0⋅001) and a treatment by type interaction (*P* = 0⋅002) for BF. *Post hoc* analyses indicate that the actual change in BF was less than theoretically expected for ADD (0⋅1 ± 0⋅2 *v.* 1⋅2 ± 0⋅1 %; *P* < 0⋅001) but not SUB (1⋅0 ± 0⋅3 *v.* 1⋅1 ± 0⋅1 %; *P* = 0⋅97) (Fig. 2(b)). Finally, the change in the actual BF was smaller in ADD *v.* SUB (0⋅1 ± 0⋅2 *v.* 1⋅0 ± 0⋅3; *P* < 0⋅001).

### Regression analysis

Exploratory two-way stepwise multiple regression and best subsets multiple regression approaches were utilised to determine predictors of the change in BW and BF in the two pecan groups. For BW, the two-way stepwise model and Mallow's *C_p_* indicated the best model to be the one in which the ERS and the difference score from the fat preference questionnaire were included. BIC indicated the best model to be the one in which only the ERS was included. We chose the model that included the energy report and the difference scores since these were selected by the majority of the model selection methods. When the multiple regression model was analysed, the ERS (*β*=−0⋅001; *P* < 0⋅001) and the difference score (*β*=0⋅02; *P* = 0⋅12) explained 28 % of the variability in the change in BW within the two pecan groups (full model: intercept = 0⋅30; *P* < 0⋅001). For BF, all three model selection methods determined the best model to be one which included treatment and baseline sugar intake. When the multiple regression model was analysed, treatment (*β*=0⋅82; *P* = 0⋅02) and baseline sugar intake (*β*=0⋅01; *P* = 0⋅01) explained 22 % of the variability in the change in BF (full model: intercept=−0⋅63; *P* < 0⋅001).

### Dietary intake

Average dietary intake at baseline and throughout the intervention is presented in Table 3. There were no differences in dietary intake between groups at baseline. For total EI (kcal/d), there was a significant main effect of time (*P* = 0⋅005) but no effect for treatment (*P* = 0⋅17) or a time by treatment interaction (*P* = 0⋅20). The main effect of time indicates that EI increased from baseline regardless of treatment. For the percentage of energy from carbohydrate, fat and protein, there was a significant main effect of time (*P* < 0⋅01 for all), treatment (*P* < 0⋅01 for all) and a time by treatment interaction (*P* < 0⋅001 for all). *Post hoc* analyses revealed that there was an increase in the percentage of energy from fat (*P* < 0⋅001 for both) and a decrease in the percentage of energy from carbohydrate (*P* < 0⋅001 for both) and protein (*P* = 0⋅01 and *P* = 0⋅05, respectively) within ADD and SUB, but not control, from baseline to throughout the intervention.

**Table 3.** Lifestyle factors at baseline and during the intervention

	ADD (*n* 30)				SUB (*n* 31)				Control (*n* 32)			
	Baseline		Intervention		Baseline		Intervention		Baseline		Intervention	
Dietary intake	Mean	sem	Mean	sem	Mean	sem	Mean	sem	Mean	sem	Mean	sem
Distribution of energy												
Energy (kcal)^a^	2141	143	2468	145	2161	104	2280	79	1998	108	2073	116
Kcal from carbohydrate (%)^a^^,^^b^	48	2	37*	1	49	1	38*	1	48	1	46	1
Kcal from protein (%)^a^^,^^b^	16	0⋅4	14*	0⋅5	14	0⋅5	13*	0⋅3	15	0⋅5	16	0⋅5
Kcal from fat (%)^a^^,^^b^	36	2	48*	1	36	1	47*	1	36	1	37	1
Kcal from alcohol (%)^a^	1	0⋅3	1	0⋅4	1	0⋅5	1⋅5	0⋅4	1	0⋅4	2	0⋅6
Carbohydrates												
Fibre (g)^a^	14	1	21*	1	14	2	18*	1	16	1	15	1
Sugar (g)^a^	81	6	75	5	105	12	79*	8	78	5	79	8
Fats												
MUFA (g)^a^^,^^b^	34	3	62*	4	35	3	55*	2	28	2	33	2
PUFA (g)^a^^,^^b^	19	2	33*	2	21	2	30*	1	18	2	20	1
*n*-3 FA (g)	2	0⋅5	2	0⋅2	2	0⋅4	2	0⋅1	2	0⋅6	2	0⋅3
*n*-6 FA (g)^a^	13	2	29	2	15	2	26	1	11	1	26	12
SFA (g)	30	3	31	3	28	2	28	1	30	2	28	2
Trans-FA (g)	0⋅8	0⋅1	0⋅8	0⋅1	0⋅8	0⋅2	0⋅7	0⋅1	0⋅8	0⋅1	1⋅0	0⋅2
Cholesterol (mg)	330	58	316	54	293	34	225	16	236	24	260	21
IPAQ short form												
Total PA (MET, min/week)	1647	248	1799	278	1331	296	1068	185	1116	164	1295	181
Vigorous PA (MET, min/week)	725	141	672	122	335	173	313	109	389	89	501	99
Moderate PA (MET, min/week)	336	120	574	127	285	76	290	80	246	53	327	73
Walking (MET, min/week)	556	104	559	114	711	171	464	98	481	82	468	69
Sitting time (min/d)	375	37	374	32	415	37	444	50	399	39	433	32
Activity EE (kcal/d)	289	44	333	55	254	50	208	38	215	43	257	53
Fat Preference Questionnaire												
Taste score (%)	65	3	63	4	67	3	66	3	70	3	71	3
Frequency score (%)	42	4	41	4	48	4	47	3	50	3	49	4
Difference score (%)	22	3	22	3	19	3	19	3	20	2	23	3
Other variables												
Perceived Stress Scale Score^a^	14	0⋅6	12	0⋅9	15	1⋅3	14	1⋅1	12	1⋅1	11	1⋅2
Energy Report Score	–	–	−230	104	–	–	−432	107	–	–	−427	91

ADD, consumed pecans as part of a free-living diet; SUB, substituted pecans for isocaloric foods from their habitual diet; FA, fatty acid; g, gram; kcal, kilocalorie; MET, metabolic equivalent; MUFA, monounsaturated fatty acid; PA, physical activity; PUFA, polyunsaturated fatty acid; IPAQ, International Physical Activity Questionnaire; MET, metabolic equivalent.

For the fat preference questionnaire, taste and frequency scores were calculated based on the percentage of food sets in which high-fat foods were reported to ‘taste better’ and be eaten more often, respectively. Difference scores were calculated by subtracting the frequency score from the taste score. The energy report score, a measure of under- or over-reporting on food diaries, was calculated by subtracting the estimated energy intake during the study from the average energy intake reporting on food diaries during the intervention. The estimated energy intake during the study was calculated based on changes in body weight using the National Institute of Health Body Weight Planner^(21)^.

* This indicates a significant time by treatment interaction with greater changes in a group compared to the control (*P* < 0⋅05).

a This indicates a significant main effect of visit.

b This indicates a significant main effect of treatment at *P* < 0⋅05.

For dietary fibre and sugar, there was a main effect of time (*P* < 0⋅001 and *P* = 0⋅04) and a time by treatment interaction (*P* < 0⋅001 for both) but no effect of treatment (*P* = 0⋅45). The interaction was for an increase in fibre within ADD (*P* < 0⋅001) and SUB (*P* = 0⋅04) with no change in control, and a decrease in sugar within SUB only (*P* = 0⋅03) from baseline to intervention. There were significant main effects for time (*P* < 0⋅001 for both), treatment (*P* < 0⋅01 for both) and a time by treatment interaction (*P* < 0⋅001) for MUFA and PUFA intakes (g/d). The interaction was for an increase in MUFA (*P* < 0⋅001 for both) and PUFA (*P* < 0⋅01 for both) within ADD and SUB but not control. For *n*-6 fatty acids, there was a main effect of time (*P* = 0⋅001) but no treatment (*P* = 0⋅86) or time by treatment interaction (*P* = 0⋅90), indicating that the intake of *n*-6 fatty acids increased from baseline regardless of group. There were no main or interaction effects for saturated fat, trans-fat, cholesterol or *n*-3 fatty acids (ns).

### Questionnaires

Average questionnaire responses for stress, physical activity and fat preferences at baseline and throughout the intervention are presented in Table 3. There were no differences between groups at baseline for any questionnaire outcome. For perceived stress, there was a main effect of visit (*P* = 0⋅04) but no treatment effect (*P* = 0⋅15) or interaction (*P* = 0⋅47). The visit effect indicates a reduction in perceived stress from baseline regardless of treatment. For all self-reported measures of physical activity and fat preference, there were no significant main or interaction effects. Finally, the ERS, a measure of under- or over-reporting on food diaries, was not different between groups (*P* = 0⋅27).

## Discussion

There was an increase in BW regardless of treatment, which was predominately driven by the two pecan groups that were trending for an increase in BW compared to control. Although not statistically significant, the average weight gain of 0⋅9–1⋅1 kg in the pecan groups is clinically meaningful since the average annual weight gain for adults is 0⋅5–1 kg/year^(25)^. It is likely that the slight increase in BW (0⋅3 ± 0⋅2 kg) and self-reported EI (75 ± 117 kcal/d) in the control group inhibited our ability to observe differences between groups for these outcomes. The estimated theoretical increase in BW in the two pecan groups was considerably higher than actual changes in BW by approximately 2⋅2 kg, indicating a fairly large degree of compensation from the added energy content of the pecans and/or other nutrients in the nuts such as fibre, total fat, MUFA or PUFA. It is likely this partial compensation also contributed to the non-significant differences in weight change between pecan *v.* control groups. We did, however, observe increases in BF in the group receiving the isocaloric substitution instructions (SUB) only. While significant, that change in BF was less than the theoretical change in ADD (0⋅1 ± 0⋅2 *v.* 1⋅2 ± 0⋅1 %) but not SUB. There were no changes in total physical activity (MET, min/week), suggesting that any anthropometric changes were likely influenced by EI.

Based on our initial hypothesis, it was surprising that significant weight gain did not occur in the ADD group compared to the other two groups. Two previous intervention studies that directly compared the impact of different dietary practices during nut interventions showed that BW or BF increased in groups without dietary instructions, while the dietary instructions protected against these unfavourable changes^(15,16)^. It is possible that the divergence in results between those studies and the present study were due to differences in methodology. For example, the walnut intervention was 6 months in duration in the study by Njike *et al.*^(15)^; thus, the present study duration may not have been long enough to capture differences between our two pecan groups. Furthermore, the crossover study by Alper *et al.*^(16)^ involved three treatment arms that all consumed peanuts with varying degrees of dietary instructions. The lack of a true control group in that study may explain why they were able to capture differences between peanut groups. Although our BW results are not in line with these two previous studies, our findings do corroborate with the substantial epidemiological and interventional evidence that tree nuts (such as almonds and walnuts) are beneficial for weight management, even without isocaloric substitution instructions^(7,26)^.

There are several potential mechanisms for how tree nuts promote weight maintenance, despite their high energy density, which may explain our lack of significant weight gain between pecan groups *v.* control in the present study. Tree nuts are rich in protein, fibre and energy, which may prevent further food intake by inducing satiety^(27)^. We previously showed that a 7-d PUFA-rich diet (containing walnuts) improved fasting and postprandial peptide YY, a satiety hormone^(28)^. Furthermore, previous research involving 8–19 weeks of peanut or pecan consumption resulted in a 5–11 % increase in resting metabolic rate^(16,29,30)^. Finally, we know that not all the energy in some nuts is fully metabolised and absorbed^(31,32)^. The metabolisable energy (ME) of almonds^(31)^, walnuts^(32)^, cashews^(33)^ and pistachios^(34)^ is 32, 21, 16 and 5 % less than predicted by the Atwater factors, respectively. The ME of pecans has not been elucidated, but we might speculate that it is less than expected due to our observed weight maintenance. Altogether, the results of the present study may be due to a combination of increased satiety and energy expenditure and decreased absorption of the energy from pecans.

Although we did not find a significant difference in weight gain between pecan groups *v.* control, we did observe a large variation of weight change in the pecan groups (sd of 1⋅4 kg). Therefore, we conducted exploratory regression analyses to investigate predictors of changes in BW and BF during pecan interventions. The exploratory regression analyses indicated that the energy report and difference scores explained 28 % of the variability in the change in BW within the two pecan groups. A positive ERS corresponded with over-reporting on food diaries, while a negative score indicated under-reporting on food diaries. The *β* coefficient suggests that for every 1 kcal decrease in the ERS (under-reporting), the change in weight increased by an additional 0⋅001 kg when the difference score is held constant. Previous research indicates that under-reporting EI is also positively associated with dietary restraint, the tendency to restrict food^(35,36)^. Furthermore, the difference score from the fat preference questionnaire is a measure of dietary fat restraint and is also associated with standard measures of dietary restraint^(37)^. The *β* coefficient suggests that for every 1 % increase in the difference score, the change in weight increased by an additional 0⋅02 kg when the ERS is held constant. At first glance, it appears contradictory that increased dietary restraint would predict weight gain, but previous research suggests that dietary restraint may increase vulnerability to weight gain, especially in women^(38)^. Although pecans have been shown to provide a variety of health benefits^(39–41)^, a history of dietary restraint may be an important consideration before recommending daily pecan consumption, especially in high doses.

It was unexpected that BF increased in the SUB and not the ADD group. Although surprising, many other tree nut studies have also observed changes in one, but not both, of these outcomes^(16,42–44)^. Although DXA is more accurate and precise than other methods for measuring body composition^(45)^, it is still vulnerable to inaccuracies of approximately 1 %^(46)^. Therefore, it is possible that a 1⋅1 % increase in BF within SUB, or the lack of change within ADD, falls within measurement error. The free-living nature of the present study is another potential limitation, as extraneous factors such as weather and family circumstances may have influenced lifestyle behaviours that impact BW. However, the design of the present study was intentional in effort to increase the generalisability of the results. The self-report nature of the assessments for dietary intake, physical activity and stress were another limitation as they are vulnerable to under- and over-reporting. Likewise, we utilised 2-d food records instead of the standard 3-d food records at baseline to reduce participant burden. Since participants completed weekly food records once per week alternating between weekdays and weekend days, our reporting of the average intake during the intervention may over-emphasise weekend days since those days occur less frequently throughout a week. Finally, the present study was not powered or designed to detect differences between sexes or races, and the short duration of the study limits conclusions for long-term weight management.

In conclusion, daily pecan consumption (68 g/d), regardless of isocaloric substitution instruction, did not result in significant weight gain. The slight, non-significant increase in weight in the control group likely affected our ability to detect a significant change in BW in either pecan group compared to control. Although the non-significant weight gain with pecan consumption may be clinically meaningful, it was much less than the theoretical weight gain, indicating at least partial compensation for the added energy from the pecans. We did observe an increase in BF in one of the two pecan groups, although it is unclear why this occurred in the group performing the dietary substitution instructions. Future research should further investigate the ME of pecans and the impact of dietary restraint on changes in BW and BF during tree nut interventions.

## Acknowledgments

This work was supported by the Georgia Pecan Commission (No. AWD00010306).

J.A.C. and C.M.P. conceived the project, provided study oversight and provided essential reagents and materials. J.A.C. and L.L.G. developed the overall research plan and wrote the manuscript. L.L.G. conducted the research and analysed data.

There are no conflicts of interest.
